# Dental Education

**Published:** 1854-04

**Authors:** 


### DENTAL EDUCATION.

So many different views are entertained in regard to the education proper for a dentist, that it would seem a superfluous task on our part to add another to the thousand and one speculations already before the world. Still, as we have become in a measure identified with the question, silence, at this time, might be construed into a hesitancy about expressing our opinions, which we are far from feeling.

The primary question to be decided by any one really in earnest about a professional education, is simply this: What is the position I design to occupy? This being answered, the rest is soon settled. Now, what is the position which the better class of dentists have been for many years aiming at? What is their ambition? Why, the very laudable one of having their art recognized as a specialty of medicine. They claim an equality with aurists, oculists, othopœdists, and specialists generally, who have succeeded in getting themselves recognized by the medical profession.

Now, no one that we have ever heard of, pretends to rank any exclusive specialty in any science as equal to the whole science itself. The technological chemist, though he may have exhausted all existing knowledge in his department, is rated by no one, whose opinion is worth having, on a par with the chemical philosopher, whose studies and acquirements embrace the whole round of the science, and comprise the results of myriads of observations in all the specialties.

There is, however, a rank among specialists themselves, and to keep clear of any suspicion of personality, we continue our illustration from the chemical profession. Take two technological chemists, one of whom is a routinist, and the other an observer. The routinist is drilled thoroughly in all the deduced principles of his department. He understands all the manufacturing processes, and conducts his work with precision and accuracy. He may suggest improvements in the arrangement of the parts of a factory, or detect errors in the construction of the flues. He is a reliable man, and enjoys the full confidence of the master-manufacturer who employs him, and the respect of those who are under him.

The other is as well drilled as his brother chemist in all the technological deductions of chemistry, but he has gone farther. He has extended his studies into the domain of chemical philosophy. He understands the principles which underlie the formulæ, and can criticise not only the operations, but the rules which guide them. He is, therefore, prepared to take advantage of his position, and to gain that knowledge of the influence of quantity upon chemical changes, which the analyst and the laboratory chemist can never acquire. He reasons upon what he sees, detects fallacies that escape the scrutiny of the routinist, and adds to the common stock, not only of technological, but of philosophical chemistry.

Now, that these two men hold very different relations to the higher class of chemical philosophers, no one can fail to perceive. Both are specialists; but one is a specialist, and nothing else; the other touches upon the domain of philosophy, and obtains a recognition which is not accorded to his brother chemist.

Apply these remarks, these facts, to the position of the dentist. How can the medical profession, with its learning, its skill, its high position, its centuries of renown, its memories, its muster-roll of great names, its army of great intellects, before whom all men must bow in respect, how can such a profession recognize a mere mechanical pursuit as a department of its own great art?

Now, it is of no avail to attempt to reply to this by citing the ignorance of many members of this exalted profession. Plenty of asses, calling themselves doctors, there undoubtedly are, but for all that, *medicine* is not asinine. The medical schools have generally been untrue to their trust, and have overwhelmed the profession with fools, for the meanest of all mercenary considerations. The best defence that they can make is, that their graduates are somewhat better than thc horse-doctors, and the Indian-doctors, and the old women, and the Thompsonians, whom they supersede; and their honest confession that, were they to reject every man who could not spell the words of his mother-tongue correctly, to say nothing of speaking or writing tolerable English, they would be compelled to close their doors, is a melancholy admission of their inability to elevate the standard of the profession, or even to keep it up to what it was before they commenced tinkering at it. But these boobies that feel pulses, and look at tongues, and give pills all over the land, are not the medical profession. The very professors who give them their diplomas would scorn to meet them on terms of professional equality. *Their* recognition or non-recognition of anybody or anything, amounts to nothing at all. For all this paralysis in the extremities, however, the heart and brain of the medical profession are still sound. There are still profound learning, great acumen, high intellectual powers, boundless acquirements in its members, in spite of the low standard of requirements acknowledged by the colleges; so that the plea alluded to is worthless, and he who claims affiliation with this profession, comes before a tribunal fully competent to decide upon his merits, with an authority that all the world must submit to.

It is manifest, therefore, that before a specialty can pretend to a recognition from so learned a body, it must itself be learned. It must have sufficient knowledge of general science to be able to determine when its own facts can add to the common stock, and what the value of those facts is. It must, in short, have some general idea of that art to which it claims to belong. Without this, it must forever remain neglected and obscure, obtaining no respect because deserving none.

If, then, a dentist wishes only to be a mechanic, exercising his ingenuity upon the teeth, let him have a mechanic's education, and take a mechanic's rank in the scientific world. If he desires to be acknowledged as a collaborator with the physician, let him get such a knowledge of general science and literature as will qualify him for such associations. It is the height of folly for any man to struggle into a society in which he cannot maintain himself. He only pays a premium for ridicule, and offers a bribe for contempt.

